# Application of food exchange portion method in home-based nutritional intervention for elderly patients with chronic heart failure

**DOI:** 10.1186/s12872-023-03072-7

**Published:** 2023-02-10

**Authors:** Ce Zhou, Shan Wang, Xing Sun, Yuhao Han, Li Zhang, Meixia Liu

**Affiliations:** 1 Department of Cardiology, Hebei Provincial People’s Hospital, 348 Heping West Road, Hebei 050051 Shijiazhuang, China; 2 Department of Nephrology, Jinzhou People’s Hospital, Jinzhou City, 052260 China

**Keywords:** Food exchange portion, Chronic heart failure, Body fat rate, Metabolic equivalent, Visceral fat area

## Abstract

**Background:**

The home treatment of elderly patients with chronic heart failure (CHF) is often accompanied by malnutrition, which increases the risk of re-hospitalisation and affects the prognosis. Therefore, how to effectively improve the nutritional self-management of patients is a current focus of medical research. This study aims to test the effect of home-based nutritional intervention method on improving the nutritional status of elderly patients with CHF.

**Methods:**

A total of 90 hospitalised elderly patients with CHF were randomly divided into the experimental group (*n* = 45) and the control group (*n* = 45). The patients in both groups were given standardised drug therapy and their nutritional status was evaluated using a body composition analyser prior to discharge (protein, body fat percentage, visceral fat area, skeletal muscle, upper arm muscle circumference, left lower limb and right lower limb muscle mass), with the cardiopulmonary function evaluated using a six-minute walk test and the metabolic equivalents method. The control group was given general nutrition education and routine dietary guidance from cardiac rehabilitation nurses, while the experimental group was given an individualised nutrition prescription by dietitians based on the evaluation results, according to which one-to-one food exchange dietary intervention training was given until the patients mastered the process.

**Results:**

The nutritional indexes at the end of the study were significantly higher in the experimental group than in the control group and were higher than those before the intervention (*P* < 0.05). The muscle circumference of the upper arm, the muscle mass of the left lower limb and the right lower limb had no statistical significance following the intervention compared to the control group and before the intervention (*P* > 0.05). The cardiopulmonary function indexes were significantly better in the experimental group at the end of the study than before the intervention and were better than those in the control group, with statistically significant differences (*P* < 0.05), while no significant changes were observed in the control group before and after the intervention (*P* > 0.05).

**Conclusion:**

The home-based nutritional intervention method of food exchange portions can effectively improve the nutritional status of elderly patients with CHF, with the distribution of visceral fat more reasonable and the cardiopulmonary function and exercise endurance improved.

## Introduction

Chronic heart failure (CHF) is the terminal stage of heart disease caused by various aetiologies and has the characteristics of high morbidity, high readmission and high fatality rates and can seriously endanger human health. In recent years, the prevalence of CHF has been increasing among the elderly. Following hospitalisation, the patients tend to return to their families for long-term drug consolidation treatment and nutritional conditioning. However, the self-management effect among elderly CHF patients is not good. Recurrent heart failure symptoms and multiple hospitalisations are associated with malnutrition [1]. Studies have demonstrated that the incidence rate of malnutrition among elderly patients with stable CHF is 16%–67% [2], while that among elderly patients with acute CHF is 22%–90% and that among patients with severe heart failure is as high as 90%. Malnutrition is an independent predictor of poor clinical outcomes in patients [3]. Therefore, how to help elderly CHF patients based at home effectively carry out nutritional self-management to prevent the occurrence of malnutrition has become a major focus of medical research.

The food exchange method involves dividing the food items into four categories according to their source and nature, with one exchange portion determined for each type of food. The energy of each food is 376.56 kJ, and the food exchange portions can be exchanged equally. This method allows for quickly estimating and calculating the energy intake per person per day, and has the characteristics of easy operation, simple and flexible food selection and elimination of the restrictions on food types [4]. The ‘gesture’ measurement of the food exchange method refers to the comparison of different shapes (actions) and parts of the hand with various food exchanges (one or several servings) and utensils of different capacities, meaning the hand is used as an estimator for food intake, facilitating the attendant calculations. In this study, based on the relatively mature dietary intervention of the food exchange method in the nutritional intervention of diabetic patients, and under the guidance of the nutritional status assessment obtained using a body composition analyser, the nutritional intervention was administered to home-based elderly CHF patients for two months.

## Materials and methods

### Research participants

A total of 90 elderly patients with stable CHF who were treated and discharged from the Cardiology Department of Hebei Provincial People's Hospital from September 2019 to June 2021 were selected as the research participants. The patients were randomly divided into the experimental group and the control group using the random number table method, with 45 patients in each group.

The inclusion criteria included: (1) patients with hypertensive heart failure and CHF who met the diagnostic criteria of the Chinese Heart Failure Diagnosis and Treatment Guidelines 2018 [5]; (2) patients with cardiac function II to III according to the New York Heart Association’s cardiac function classification [6]; (3) patients aged ≥ 65 years, with clear consciousness, barrier-free communication, normal hand functioning and the capacity to walk by themselves or with the help of a walker; and (4) patients with heart failure with reduced ejection fraction (rEF)-type CHF. The exclusion criteria included: (1) patients with a history of diabetes or other metabolic diseases; (2) patients with concomitant infection; (3) patients undergoing hormone therapy; (4) patients experiencing heart failure recurrence during the study period; (5) patients experiencing failure of organs other than the heart; (6) patients presenting with a malignant tumour; and (7) patients with a pacemaker (affecting body composition and bioelectrical impedance analysis [BIA]). This study was conducted with approval from the Ethics Committee of Four departments of cardiovascular medicine and in accordance with the declaration of Helsinki. Written informed consent was obtained from all participants.

### Research methods

Both groups of patients were treated with conventional treatment for heart failure, while at the same time, a cardiac rehabilitation manual compiled by the department based on the British Cardiac Rehabilitation Manual (The Heat Manual) was issued. The manual included six parts, with sections on drugs, sports, nutrition, emotional sleep, self-care and follow-up assessments [7]. The cardiac rehabilitation nurses provided nutrition-related education for the patients based on the nutrition content in the cardiac rehabilitation manual along with food and dietary guidance, including in terms of low-salt, low-fat, vitamin- and protein-rich diets, eating less frequently, daily intake and output calculations and weight monitoring. After being discharged from the hospital, a follow-up was conducted over the telephone once every two weeks to answer any questions raised by the patients in a timely manner and to encourage the patients to carry out self-nutrition management. Body composition analysis and a six-minute walking test (6MWT) were conducted in both groups one day before and two months following discharge.

#### Nutritional intervention methods in the experimental group

In the food exchange method, the daily food intake required by the patient is measured according to the ‘food exchange portion’ of cooked food rather than the traditional raw weight (in grammes), with the portions formulated by the nutritionist. Here, the different parts and shapes (actions) of the hand serve as the estimator of the food exchange portion, which corresponds to the cooked food volume of various foods and includes a clenched fist, a half-clenched fist and the palm. The tableware used included one ceramic spoon (measures the vegetable oil), one 4-inch rice bowl (320 ml) (measures the staple food), and one salt control spoon (2 g). The patients were then given simple measurement instructions, which included one fist (one hand) of fruit, two fists (two half fists) of vegetables, three spoons of vegetable oil, four fists of staple food, five palms of fish (or five small fish) International) protein, and six fists of water (about 1500 ml water) per day.

A dietary intervention group was then established, which included full-time nutritionists, competent doctors and cardiac rehabilitation nurses to formulate individualised nutritional prescriptions according to the patient's condition, body composition analysis data and 6MWT distance, as well as their activity intensity and daily nutrition intake following discharge. The total calories and the ratio of the three major nutrients were then recorded, with the daily caloric energy supply set at 20–30 kcal/kg according to the daily activity of the individual, of which protein accounted for 10%–14% of the total energy at 1.2–1.5 g/kg per day [8]. The food items of the nutrition prescription were then converted into a specific food exchange portion and the unit of human caloric requirement changed from kcal to food exchange portion. Each food exchange portion contained 90 kcal of energy.

The nutritionist then had a one-to-one consultation with the patients in hospital face to face or through web software like wechat to discuss the nutrition prescription of the food exchange intervention, including in terms of the daily food intake of cereals, potatoes, proteins, vegetables, nuts, fruits, oils and fats, as well as how the six categories of food are allocated to three meals, and how the food items are interchanged.

The nutritionists then guided the patient in how to use their hands as a food exchange portion estimator, demonstrating the different postures and parts of the hand that correspond to various food exchange portions. Foods of the same type are interchangeable, while foods of different types are not. Each patient was asked to practice measuring the volume of different foods of the same caloric value until they mastered the required number of food exchanges and could measure the food exchange portions proficiently.

### Research tools and indicators

#### Nutritional testing tools and indicators

In this study, a body composition analyser (INBODY S10, Biospace Co., Ltd., Korea) was used to measure the body composition of the patients, which was subsequently used for the determination of nutritional status via the BIA method. Prior to the measurement, the patient was asked to adopt a sitting or lying position, with their upper limbs abducted by 15° and the lower limbs naturally separated, while the electrode clips were fixed on the thumb, middle finger and heel of both sides. Before placing the electrodes, the skin was cleaned with 75% ethanol to reduce any skin contact resistance. The patients were strictly forbidden to eat, drink, speak or move during the test. Before commencing the measurement, the patient's hospital number, gender, age, height, weight and other information were recorded.

Body composition analysis can be used to measure protein mass (kg), skeletal muscle mass (kg), upper arm muscle circumference (cm), left lower limb muscle mass (kg), right lower limb muscle mass (kg), body fat (kg), body fat percentage (%), visceral fat area (cm^2^) and other nutritional indicators. The measurements in this study were all performed by the same nutritionist from the cardiac rehabilitation team of our department.

#### Cardiopulmonary function testing tools and indicators

In this study, the 6MWT test system (Doctor Walker 6-min walk detection and analysis system WOKE-360, Wuhan Qingyi Yunkang Medical Equipment Co., Ltd.) was used to measure the cardiopulmonary function of the patients. The test involved asking the patients to walk along a straight 20–30-m closed corridor at the fastest possible speed to test their walking distance within a six-minute period. During the test, the patient wore a remote electrocardiogram monitoring and blood oxygen saturation monitoring system, and the patient's cardiopulmonary function and exercise tolerance were determined according to their walking distance (m) and the changes in heart rate, blood oxygen level and breathing during the test. The cardiopulmonary function of the patients was divided into four grades; the lower the grade, the worse the cardiopulmonary function. This test was carried out in the allocated test site of the Cardiac Rehabilitation Center of Hebei Provincial People's Hospital and was completed by the same rehabilitation technician from the cardiac rehabilitation team of our department. The following cardiopulmonary function grades applied: grade 1: < 300 m, grade 2: 300–374 m, grade 3: 375–449.5 m and grade 4: > 450 m.

A metabolic equivalent (MET) presents a quantitative value of exercise endurance, which pertains to the maximum exercise load an individual can bear. The larger the value, the greater the exercise load the individual can bear and the stronger the exercise ability.

### Statistical processing

In this study, SPSS 22.0 statistical software was used for the data processing and analysis. The enumeration data were expressed as a percentage (%), with inter-group comparisons made using a χ^2^ test, while the measurement data were expressed in terms of mean and standard deviation (x ± s), with the inter-group comparisons made using an independent sample *t*-test. A *P*-value of < 0.05 was considered to be statistically significant.

## Results

### Baseline data

In the experimental group, there were 27 male patients and 18 female patients, with an age range of 65–82 years. In terms of disease types, 29 patients had hypertensive heart failure and 16 had coronary heart disease, with 19 patients having cardiac function II and 26 having cardiac function III. The EF was 42–48%, with an average of (45.21 ± 1.03)%. The control group consisted of 25 male patients and 20 female patients, with an age range of 65–84 years. In terms of disease type, 26 patients had hypertensive heart failure and 19 had coronary heart disease, with 21 patients having cardiac function II and 24 having cardiac function III. The EF was 42–48%, with an average of (44.98 ± 0.99)%. There was no significant difference in terms of gender, age, disease type or cardiac function classification between the two groups (*P* > 0.05) (Table 1).

**Table 1:** Comparison of background variables between the two groups (N = 90)

Group	Cases	Age (x ± s, Year)	GenderMale/Female	Hypertensive heart failure n (%)	Coronary heart disease and heart failure (%)	Cardiac function class II n (%)	Cardiac function grade III n (%)
experimental group	45	68.15 ± 12..42	27/18	29 (64.44)	16 (35.562)	19 (42.22)	26 (57.78)
control group	45	70.35 ± 9.28	25/20	26 (57.78)	19 (42.22)	21 (46.67)	24 (53.33)
X2 (t) value		− 0.952	0.182	0.435	0.421	0.180	0.116
*P* value		0.344 > 0. 05	0.670 > 0. 05	0.517 > 0. 05	0.510 > 0. 05	0.671 > 0. 05	0.538 > 0. 05

### Nutritional states after intervention

There was no significant difference in nutrition-related indexes between the two groups prior to the intervention (*P* > 0.05). After two months of intervention, the nutritional indicators in the experimental group changed significantly, with the decrease in body fat and body fat rate significantly higher than before the intervention and higher than those in the control group; the differences were statistically significant (*P* < 0.05). The degree of reduction in visceral fat area was significantly higher in the experimental group than in the control group, with statistical significance (*P* < 0.01), while the increase in protein quality and skeletal muscle mass was also significantly higher in the former than in the latter, again with statistical significance (*P* < 0.05). Based on these results, we suggested that the nutritional states of experimental group after intervention was improved significantly compared with the control group. The comparison before intervention was not statistically significant (*P* > 0.05) (Table 2).

**Table 2:** Comparison of nutritional states related variables between the two groups before and after intervention (N = 90)

Group	Cases	Protein (kg)		Body fat (kg)		Percentage of body fat (%)		Visceral fat area (cm^2^)	
		Before intervention	After intervention	Before intervention	After intervention	Before intervention	After intervention	Before intervention	After intervention
experimental group	45	7.08 ± 2.58	9.04 ± 2.64 a	22.48 ± 11.35	17.6 ± 6.87 a	31.13 ± 8.84	23.74 ± 6.97 a	118.5 ± 13.2	86.8 ± 11.7 b
control group	45	7.64 ± 2.03	7.83 ± 2.76 c	21.98 ± 10.30	21.53 ± 8.78 c	30.26 ± 7.61	29.45 ± 9.61 c	121.6 ± 12.9	118.1 ± 9.8 c
T value		− 1.144	2.125	0.219	− 2.365	0.500	− 3.227	− 1.127	− 11.560
*P* value		0.256	0.036	0.827	0.020	0.618	0.002	0.263	0.000

Group	Cases	Skeletal muscle (kg)		Upper arm muscle circumference (CM)		Left lower limb muscle (kg)		Right lower limb muscle (kg)	
		Before intervention	After intervention	Before intervention	After intervention	Before intervention	After intervention	Before intervention	After intervention
experimental group	45	17.98 ± 5.26	21.89 ± 7.61a	21.48 ± 2.21	22.07 ± 3.61 c	6.54 ± 2.12	7.17 ± 3.41 c	6.74 ± 1.79	7.43 ± 2.52 c
control group	45	18.64 ± 6.02	19.27 ± 2.80 c	22.17 ± 3.53	22.39 ± 2.41 c	7.01 ± 2.97	6.95 ± 3.13 c	7.24 ± 2.39	7.16 ± 2.43 c
T value		− 0.554	2.168	− 1.111	− 0.495	− 0.864	0.319	− 1.123	0.517
*P* value		0.581	0.033	0.269	0.622	0.390	0.751	0.264	0.606

“a” refers to the comparison before intervention (*P* < 0 05, “b” is the comparison before intervention (*P* < 0.05) 01, “c” is the comparison before intervention (*P* > 0 05)

### Cardiopulmonary function indicators

There was no significant difference in 6MWT walking distance (m) and METs between the two groups before the intervention (*P* > 0.05). After two months of intervention, the 6MWT walking distance and METs of the patients in the two groups had increased, with the improvement in the experimental group significantly higher than that in the control group; the differences were statistically significant (*P* < 0.01) (Table 3).

**Table 3:** Comparison of cardiopulmonary function related variables between the two groups before and after intervention (N = 90)

Group	Cases	6MWT walking distance (m)				Metabolic equivalent (MetS)			
		Before intervention	After intervention	T value	*P* value	Before intervention	After intervention	T value	*P* value
experimental group	45	371.66 ± 58.16	442.53 ± 54.36	− 5.972	0.000	3.36 ± 1.81	4.78 ± 1.15	− 4.442	0.000
control group	45	384.68 ± 47.21	396.39 ± 48.63	− 1.159	0.249	3.78 ± 1.65	3.84 ± 0.81	− 0.219	0.827
T value		− 1.166	4.244			− 1.150	4.483		
p value		0.247	0.000			0.253	0.000		

## Discussion

Home-based food exchange nutritional interventions can effectively improve the malnutrition status of elderly patients with CHF. Such patients are prone to malnutrition, aggravating the disease due to its long course and to gastrointestinal blood stasis, leading to dysfunction in nutrient absorption and intake. In fact, malnutrition has become an important factor for the recurrence of symptoms in CHF patients, meaning home-based nutritional intervention for elderly CHF patients is crucial [9]. At present, there are no relevant clinical guidelines or general consensus among experts regarding dietary nutrition for patients with cardiovascular disease or elderly patients with CHF. While the concept of nutritional intervention has attracted the attention of the majority of clinical cardiovascular medical staff, it mostly focuses on the evaluation of nutritional status and simple dietary guidance, such as limiting water and sodium intake, adequate supplementary vitamin intake, eating small meals frequently and avoiding fullness, and there remains no specific nutritional intervention.

The ‘Food Exchange Gesture Measurement Rule’ was proposed by the Canadian Diabetes Association Clinical Practice Guidelines Expert Committee for dietary education in patients with diabetes [10], with the rule modified by Fang Yuewei according to the body structure and the dietary habits of Chinese residents. The operation of the guidelines is simple and easy to master and is highly suitable for elderly patients, with the method also used in clinical diabetic patients, often with remarkable effects [11–13]. In our hypothesis, we thought that the Food Exchange intervention method could be close related to the nutrition status to elderly patients with CHF, and it could improve the nutritional status. In this study, an individualised nutritional prescription was formulated for each patient and transformed into a form of the food exchange method, with special attention paid to the eating habits of the elderly in China. Among this population, the meals are mostly light and easy to digest, and the general protein intake is largely insufficient. Meanwhile, a high intake of carbohydrates leads to an accumulation of fat in the body. The nutritional prescription thus included a minimum of five servings of animal and plant protein per day.

The results indicated that after two months of intervention, the protein content and skeletal muscle mass were significantly higher in the experimental group than in the control group, while the reduction in body fat and body fat rate was also significantly higher in the former than in the latter. The visceral fat area particularly decreased significantly and tended to be more rationalised. Overall, the results indicated that the patients in the experimental group had effectively completed the set food exchange nutrition intervention and that their nutritional status had been greatly improved. These findings meet the expectation of our hypothesis. In our study design, we also hypothesized that the intervention method could have the close relationship with cardiovascular disease. What's similar is that Nie Qiuping, Li Changyan and others believe that the analysis of body composition can reflect the nutritional status of patients with heart failure more objectively and can accurately reflect the content and distribution of body fat in patients [14–16]. Meanwhile, the existing literature reports that the body fat rate is better than the body mass index for assessing the cardiovascular risk factors in patients with CHF [17]. Elsewhere, various studies have revealed a strong link between body fat content and cardiovascular factors and have reported that abnormal fat distribution is a risk factor for cardiovascular disease independent of obesity [18, 19]. Therefore, the intervention method presented herein can effectively reduce the incidence of cardiovascular risk to a certain extent on the premise of improving the nutritional status of elderly patients with CHF.

Furthermore, the home-based nutritional intervention food exchange method can improve the cardiopulmonary function and the exercise tolerance among elderly patients with CHF. In this study, CHF patients aged ≥ 65 years were recruited. It has been reported that an age of > 65 years is an independent risk factor for malnutrition in CHF patients [20, 21]. At home, elderly CHF patients mostly rely on bed rest, partake in less scientific aerobic exercise and tend to have different degrees of malnutrition, resulting in a general decrease in cardiopulmonary function and exercise tolerance. In this study, both the cardiopulmonary function and exercise tolerance of the patients in the two groups were low prior to the intervention. However, after two months of nutritional intervention, the 6MWT walking distance and the METs were significantly higher in the experimental group than in the control group, and the cardiac function was significantly improved. The endurance level was also significantly enhanced, indicating that, based on the premise of effectively improving the nutritional status of the patient, the food exchange method provides sufficient energy for the myocardial contraction of CHF patients, thereby improving the myocardial contractility and improving the patient's cardiopulmonary function and exercise endurance. Overall, the quality of life of the patients was improved to varying degrees which is same as our expected in hypothesis.

In this study, the upper arm muscle circumference and the left lower extremity and right lower extremity muscle mass of the patients increased in the experimental group and were higher compared to those in the control group, while there was no statistical significance. In short, the effect was insufficient. This suggests that in the future, under the premise of strengthening the nutrition for elderly CHF patients at home, attention must be paid to the formulation of the exercise prescriptions and the guidance for active and passive exercise, with home-based elderly CHF patients encouraged to perform an appropriate amount of aerobic exercise to improve the muscle content of their limbs and to further improve their cardiopulmonary function and exercise endurance.

## Conclusion

The food exchange method is easy to grasp, easy to operate and easy to master, and is highly suitable for home-based elderly CHF patients. This study confirmed that the intervention method can improve the nutritional status of patients, as well as their cardiopulmonary function and exercise tolerance. In the absence of standardised nutritional intervention guidelines for CHF patients, it is believed that the food exchange method can be applied and promoted in elderly CHF patients based at home.

However, this study involves a number of limitations. First, the participants were all home-based elderly CHF patients who were in stable condition following discharge and there was no obvious water and sodium retention or symptoms of heart failure, while the nutritional prescription was relatively fixed. Second, the haemoglobin, prealbumin and albumin levels were not measured following the intervention to assess whether the patient was anaemic or had a strong inflammatory response. Finally, the intervention time was comparatively short at two months. In future research, nutritional interventions for patients with different stages of heart failure progression will be examined.

## Data Availability

All data generated or analyzed during this study are included in this article.
